# Clinical and epidemiological characteristics of individuals resistant to *M. tuberculosis* infection in a longitudinal TB household contact study in Kampala, Uganda

**DOI:** 10.1186/1471-2334-14-352

**Published:** 2014-06-27

**Authors:** Ningning Ma, Sarah Zalwango, LaShaunda L Malone, Mary Nsereko, Eddie M Wampande, Bonnie A Thiel, Brenda Okware, Robert P Igo, Moses L Joloba, Ezekiel Mupere, Harriet Mayanja-Kizza, W Henry Boom, Catherine M Stein

**Affiliations:** 1 Department of Medicine, Case Western Reserve University, 2103 Cornell Rd, Wolstein Research Building room 1316, Cleveland, OH 44106, USA; 2 Department of Epidemiology & Biostatistics, Case Western Reserve University, 2103 Cornell Rd, Wolstein Research Building room 1316, Cleveland, OH 44106, USA; 3 Uganda – CWRU Research Collaboration, Makerere University and Mulago Hospital, Kampala, Uganda; 4 College of Health Sciences, Makerere University and Mulago Hospital, Kampala, Uganda

**Keywords:** Transmission risk factors, Latent Mtb infection, Exposure, Household characteristics, PPD test

## Abstract

**Background:**

Despite sustained exposure to a person with pulmonary tuberculosis (TB), some *M. tuberculosis* (Mtb) exposed individuals maintain a negative tuberculin skin test (TST). Our objective was to characterize these persistently negative TST (PTST-) individuals and compare them to TST converters (TSTC) and individuals who are TST positive at study enrollment.

**Methods:**

During a TB household contact study in Kampala, Uganda, PTST-, TSTC, and TST + individuals were identified. PTST- individuals maintained a negative TST over a 2 year observation period despite prolonged exposure to an infectious tuberculosis (TB) case. Epidemiological and clinical characteristics were compared, a risk score developed by another group to capture risk for Mtb infection was computed, and an ordinal regression was performed.

**Results:**

When analyzed independently, epidemiological risk factors increased in prevalence from PTST- to TSTC to TST+. An ordinal regression model suggested age (p < 0.01), number of windows (p < 0.01) and people (p = 0.07) in the home, and sleeping in the same room (p < 0.01) were associated with PTST- and TSTC. As these factors do not exist in isolation, we examined a risk score, which reflects an accumulation of risk factors. This compound exposure score did not differ significantly between PTST-, TSTC, and TST+, except for the 5–15 age group (p = 0.009).

**Conclusions:**

Though many individual factors differed across all three groups, an exposure risk score reflecting a collection of risk factors did not differ for PTST-, TSTC and TST + young children and adults. This is the first study to rigorously characterize the epidemiologic risk profile of individuals with persistently negative TSTs despite close exposure to a person with TB. Additional studies are needed to characterize possible epidemiologic and host factors associated with this phenotype.

## Background

Tuberculosis (TB) is an infectious disease caused by *Mycobacterium tuberculosis* (Mtb). There are an estimated 8 million new cases and 2 million deaths from TB annually. Uganda is one of the world’s 22 highest burden countries with TB, with an annual risk of infection of 3% and annual incidence of new smear positive TB cases of 9.2 per 1000 in an urban setting [1].

Transmission of Mtb requires repeated close contact with someone who has active pulmonary TB and the likelihood of transmission increases with increasing levels of contact. Therefore, it is not surprising that markers of close contact, such as crowded quarters, urban living, and lower socio-economic status, are associated with acquisition of Mtb [2,3]. Clinical characteristics of the person with pulmonary TB that increase the risk of contacts becoming infected include cavitary disease, sputum smear grade, and extent of disease based on x-ray [4-8].

The pathogenesis of TB follows a two-stage process [8]. Infection is the first stage, where, Mtb infection is contained but not eliminated by the innate and adaptive immune response. The second stage consists of progression from infection to active disease, and presents with signs and symptoms caused by the increasing mycobacterial load and the host response; 5-10% of individuals infected with Mtb develop clinical disease. Interestingly, a small percentage of individuals in TB-endemic settings never demonstrate evidence of latent Mtb infection despite persistent and repeated exposure. These individuals consistently test negative by the tuberculin skin test (TST) and/or interferon-γ response assay [9]. Persistently TST negative (PTST-) individuals may be (relatively) resistant to Mtb infection, and little is known about them [10]. Previous population-based and case-contact studies have identified epidemiological risk factors for infection [2,11] but have not characterized PTST- individuals specifically. A few studies in healthcare settings have identified PTST- individuals [12-14] but these studies were limited in their duration of follow-up and sample size, and because they were conducted in health care settings, not generalizable to a population-based setting. Though we have examined immunological and genetic correlates of the PTST- phenotype [9,15], these individuals have not been thoroughly characterized epidemiologically. This study examines well-characterized risk factors for Mtb infection to determine if specific epidemiologic factors explain the PTST- phenotype, and to assess whether they are simply less likely to acquire Mtb infection.

## Methods

### Study population

The study was approved by University Hospitals Institutional Review Board at University Hospitals Case Medical Center in the United States and the National HIV/AIDS Research Committee and Uganda National Council for Science and in Uganda, and all participants provided informed consent [2]. The household contact study [16] started enrolling culture-confirmed TB patients and their households in Kampala, Uganda in April 2002, and this analysis includes patients enrolled through January 1, 2008 and followed through December 2010. An index case was defined as the first identified culture-confirmed TB case in the household. Most index cases presented with TB to the Uganda National Tuberculosis and Leprosy Program treatment center at the Old Mulago Hospital in Kampala, Uganda. Some participants also came to the study after hearing about the study via other sources. Home visits were made within 2 weeks of index case’s diagnosis, and household contacts were recruited into the study. A household contact was defined as an individual who had resided in the household for at least 7 consecutive days during the 3 months prior to diagnosis of TB in the index cases. The analyses presented here excluded individuals who had TB in the past, were diagnosed with active TB disease at the time of enrollment, or who developed TB during the course of the study. Index cases and all other active TB cases were provided standard treatment regimens (except in rare cases of multidrug resistant TB when appropriate second line treatment was given). Patients were monitored clinically and repeat sputum samples obtained at 1, 2, 5, and 9 months after treatment initiation. After the initial evaluation, participants were evaluated at 3, 6, 12, and 24 months for active TB and with repeat TST if their first and subsequent TST remained negative. All individuals were monitored clinically and if signs and symptoms of TB developed, evaluated as TB suspects. Tuberculin skin testing was done using 5 TU of purified protein derivative (PPD). HIV status was determined by serology.

### Data

Each participant received a full clinical examination, including overall health and symptom survey, chest x-ray, mycobacterial smear, and TST. Sputum smear was classified based on WHO criteria. Latent Mtb infection (TST+) was defined as a reading of at least 5 mm of induration in response to PPD in children less than 5 years old or HIV-infected individuals, and 10 mm in all other individuals, at the time of enrollment [17,18]. TST conversion (TSTC) was defined as a positive TST that occurred after a negative TST with at least a 6 mm increase in induration [15,19]. TST + persons were defined as those with a positive TST at enrollment. Vaccination by Bacillus Calmette-Guerin (BCG) was determined by the presence of BCG scar. Extensive epidemiologic and clinical data were collected on individual risk factors, proximity to the index case, and characteristics of the household [2]. Mycobacterial lineage of the TB index case was identified as described by Wampande et al. [20] using the phylogenetic groupings proposed by Gagneux [21].

### Statistical analysis

Age categories were designated based on previous examination of clinically and immunologically defined groupings [22]. Comparisons were first made across the three clinical groups (PTST-, TSTC, and TST+), then by contrasting PTST- and TSTC versus TST + (baseline TST status comparison), and by PTST- to TSTC and TST + (PTST- vs. ever becoming TST positive) persons. Categorical variables were analyzed using the χ^2^ test; because all continuous variables were skewed, they were analyzed using the Mann–Whitney U test. Multiple testing correction was done to account for 22 variable comparisons × 2 functionally independent χ^2^ tests, resulting in an adjusted α = 0.0011. Nominal *P*-values are provided in the Tables, with tests significant after correction for multiple testing indicated with asterisks (*). SPSS version 20 was used.

### Ordinal regression

Multivariable logistic regression analyses, with baseline TST status as one outcome and longitudinal TST status (PTST-) as the other were initially conducted. However, we found that the same predictor variables were significantly associated with both outcomes (data not shown). Thus, we conducted an ordinal regression analysis to provide a multivariable analysis to identify which variables from the univariate analyses remained significant while controlling for the others. Logistic regression was applied to an ordinal response variable with order PTST- < TSTC < TST+, using a proportional-odds model as implemented in the VGAM package for R [23]. The model fit logit(Pr(*y* ≤ *j*)) = α + **β***x* for *j* = 1 (PTST-), 2 (TSTC). Validity of the proportional odds assumption was tested by a likelihood ratio test comparing models with and without the constraint that the odds ratio corresponding to each covariate was the same at each level of the response. A set of optimal covariates was obtained via forward stepwise selection, using *P* = 0.1 as the threshold for entry.

### Risk score

Mandalakas et al. [11] developed a risk score for latent Mtb infection in children that included 10 variables that increased the likelihood of transmission, and consisted of clinical characteristics and proximity to the index case. While the ordinal regression illustrates the significance of individual risk factors in the context of others, this risk score captures an additive accumulation of risk factors. All but one of the variables included in their 10-point score were collected as part of our study (we did not ask “is the index case the child’s primary caregiver?”) (see Additional file 1 for complete list). We evaluated this risk score and conducted statistical comparisons described above, stratified by age given its established confounding effect (Additional file 1: Tables S3 and S4). Mandalakas et al.’s risk score was developed for children, so we modified it for adults. Instead of including the mother as index case as risk category, we used the spouse of index case, and instead of the index case being the individual’s primary care giver, we included whether the adult was the index case’s primary caregiver (see Additional file 1). Thus, the score for adults went up to 10, and we retained this greater variability to increase the informativity of the score. The risk score was analyzed both as a categorical and quantitative variable. Mandalakas et al. proposed that a score ≥ 6 out of 10 was considered “high risk”; since our pediatric score only ranged to 9, we considered children with scores ≥ 5 and adults with scores ≥ 6 to be “high risk”.

## Results

Data were collected on 1318 individuals from 454 households from April 2002 to January 2008, in which 1210 household contacts had at least one year (12 months) of TSTs in individuals who were TST negative at baseline and did not develop culture-confirmed TB during two years of follow-up; 900 (74.4%) were TST + at baseline, 168 (13.8%) converted their TST, and 142 (11.7%) were persistently TST- (PTST-). The remaining 108 individuals were TST- at baseline but did not have follow-up TSTs (TST follow-up rate of 91.8%), and thus were excluded from this analysis. When individuals that were lost to follow-up were compared to those in the analysis dataset, the only difference was in the 5–15 age group (p = 0.008), where 11.9% were lost to follow-up, compared to less than 10% for the other age groups (data not shown).

### Comparison of epidemiologic risk factors for PTST-, TSTC and TST + contacts

We found that there were no significant differences in the distributions of sex, HIV status, or presence of BCG scar across the 3 clinical groups (Table 1). There was a significant difference in the distribution of age groups across the 3 clinical groups, which was most pronounced for the 5 to 15 age group, in which 45.8% of PTST- were 5 to 15, compared to 28.0% and 26.3% of this age group in the TSTC and TST + categories (*P* < 0.0005). PPD induration at baseline was also significantly different across groups (*P* < 0.0005). To further examine this difference between PTST- and TSTC, we examined the proportion of HIV-negative individuals older than 5 years of age with TST induration greater than 5 mm (Additional file 1: Table S1), and found that there was a significantly greater proportion of TSTC with this more conservative TST cutoff. However, the more stringent 10 mm cutoff is more appropriate in this TB-endemic setting [18], given the potential for cross-reactivity with BCG vaccination and resulting misclassification, so the 10 mm cutoff was used for all further analyses.

**Table 1:** Population demographics

	**PTST- (N,% of PTST-)**	**TSTC (N,% of TSTC)**	**TST + (N,% of TST+)**	**Total**	**Comparison across 3 groups**	**Comparison of PTST- vs. TSTC + TST+**	**Comparison of PTST- and TSTC vs. TST+**
**Age at baseline (years)**							
2 years or less	19 (13.4%)	22 (13.1%)	103(11.4%)	144(11.9%)			
>2 and equal to 5	27(19.0%)	24(14.3%)	93(10.3%)	144(11.9%)			
>5 or less than 15	65 (45.8%)	47 (28.0%)	237 (26.3%)	349 (28.8%)			
>15	31 (21.8%)	75 (44.6%)	467 (51.9%)	573 (47.3%)			
All	142	168	900	1210	< 0.0005*	<0.0005*	< 0.0005*
**Sex**							
Female	73 (51.4%)	98 (58.3%)	488 (54.2%)	659 (54.5%)			
Male	69 (48.6%)	70 (41.7%)	412 (45.8%)	551 (45.5%)			
All	142	168	900	1210	0.456	0.437	0.775
**HIV status**							
Negative	127 (91.4%)	157 (96.9%)	801 (92.5%)	1085 (93%)			
Positive	12 (8.6%)	5 (3.1%)	65 (7.5%)	82 (7.0%)			
All	139	162	866	1167	0.095	0.430	0.277
**Presence of BCG scar**							
No	27 (19.0%)	32 (19.0%)	184 (20.4%)	243 (20.1%)			
Yes	93 (65.5%)	117 (69.6%)	627 (69.7%)	837 (69.2%)			
Uncertain	22 (15.5%)	19 (11.3%)	89 (9.9%)	130 (10.7%)			
All	142	168	900	1210	0.381	0.151	0.254
**PPD at baseline (mm)**^**1**^							
Mean (SD)	0.30 (1.153)	1.87 (3.238)	15.91 (3.605)		N/A	N/A	N/A

PTST- = Persistently negative tuberculin skin test; TSTC = Tuberculin skin test converter; TST + = tuberculin skin test positive at baseline.

*indicates *P* value that is significant after multiple testing correction.

^1^Statistical comparisons of these three groups must account for age group; see further detail in Additional file 1.

Factors associated with transmission of Mtb and increased risk for Mtb infection (eg. share room/bed with index case) generally differed across all 3 clinical groups, with a gradual increase of the high-risk category from PTST- to TSTC to TST+, though not all comparisons achieved statistical significance after multiple testing correction (Tables 2 and 3). Characteristics associated with small homes, such as number of windows and number of rooms, were less common for PTST- compared to the other groups, though there was a reverse trend in number of people per room (Table 2). Muzigos, which are multifamily housing units with one or two rooms per family [2], were less common for individuals who were TST negative at baseline compared to those who were TST + at baseline (*P* = 0.001); this may represent poorer socioeconomic status and/or close living quarters. Clinical characteristics of the index case, such as presence of cavitary disease and increased growth of Mtb on smear, followed a similar trend (Table 3), with increasing severity of TB disease in the index case more common in TSTC and TST+. There was a significantly different distribution in relationship to the index case in individuals who were baseline TST negative versus TST positive (*P* = 0.003); there were more spouses or parents of the index case among TST + persons than among PTST- and TSTC (Table 2). There was no association between Mtb lineage in the index case and clinical group. In addition, we divided the PTST- group into those who were TST- at 12 months but lost to follow-up prior to the 24 month visit, versus those we were TST- at 24 months and found no differences between groups (Additional file 1: Table S2).

**Table 2:** Individual epidemiological measures

	**PTST-**	**TSTC**	**TST+**	**Total**	**Statistical comparison across 3 groups**	**Statistical comparison of PTST- vs. TSTC + TST+**	**Statistical comparison of PTST- + TSTC vs. TST+**
**Share bed with index case**							
No	122 (85.9%)	140 (83.3%)	698 (77.6%)	960 (79.3%)			
Yes	20 (14.1%)	28 (16.7%)	202 (22.4%)	250 (20.7%)			
All	142	168	900	1210	0.028	0.039	0.009
**Share room with index case**							
No	65 (45.8%)	83 (49.4%)	297 (33.0%)	445 (36.8%)			
Yes	77 (54.2%)	85 (50.6%)	603 (67.0%)	765 (63.2%)			
All	142	168	900	1210	<0.0005*	<0.0005*	<0.0005*
**Share meals with index case**							
No	14 (9.9%)	16 (9.5%)	65 (7.2%)	95 (7.9%)			
Yes	128 (90.1%)	152 (90.5%)	835 (92.8%)	1115 (92.1%)			
All	142	168	900	1210	0.380	0.344	0.166
**Type of house (muzigo vs other)**							
Not Muzigo	75 (52.8%)	95 (56.9%)	397 (44.4%)	567 (47.1%)			
Muzigo	67 (47.2%)	72 (43.1%)	497 (55.6%)	636 (52.9%)			
All	142	167	894	1203	0.004	0.148	0.001*
**Frequency of contact with index case**							
<1 day/wk	1 (0.7%)	2 (1.2%)	19 (2.1%)	22 (1.8%)			
1-3 day	6 (4.2%)	7 (4.2%)	38 (4.2%)	51 (4.2%)			
4-6 days	7 (4.9%)	5 (3.0%)	24 (2.7%)	36 (3.0%)			
daily	129 (90.1%)	153 (91.1%)	816 (90.7%)	1097 (90.7%)			
Total	142	167	897	1206	0.740	0.536	0.593
**Time spent with index case (hours)**							
<1	3 (2.1%)	5 (3.0%)	19 (2.1%)	27 (2.3%)			
2-6	17 (12.1%)	16 (9.5%)	78 (8.8%)	111 (9.3%)			
7-12	70 (49.6%)	87 (51.8%)	511 (57.5%)	668 (55.8%)			
13-18	29 (20.6%)	38 (22.6%)	162 (18.2%)	229 (19.1%)			
>18	22 (15.6%)	22 (13.1%)	118 (13.3%)	162 (13.5%)			
Total	141	168	888	1197	0.659	0.339	0.539
**Number of windows in house**							
Mean (SD)	2.6 (2.773)	2.64 (2.475)	1.87 (2.056)		<0.0005*	< 0.0005*	0.029
**Number of rooms in house**							
Mean (SD)	3.44 (3.183)	3.26 (2.541)	2.70 (2.383)		<0.0005*	< 0.0005*	0.041
**# People in household/room**							
Mean	7.80 (5.235)	6.76 (4.129)	6.10 (3.672)		<0.0005*	< 0.0005*	< 0.0005*
**Number of TB cases in home**							
Mean	1.67 (0.98)	1.66 (0.867)	1.61 (.896)		0.985	0.363	0.799
**Relationship to index case**							
Spouse	6 (4.2%)	10 (6.0%)	122 (13.6%)	138 (11.4%)			
Parent	3 (2.1%)	5 (3.0%)	48 (5.3%)	56 (4.6%)			
Sibling	23 (16.2%)	22 (13.1%)	113 (12.6%)	158 (13.1%)			
Avuncular	24 (16.9)	29 (17.3%)	128 (14.2%)	181 (15.0%)			
Child	59 (41.5%)	66 (39.3%)	325 (36.1%)	450 (37.2%)			
Grandparent/Child	3 (2.1%)	4 (2.4%)	18 (2.0%)	25 (2.1%)			
Unrelated	21 (14.8%)	27 (16.1%)	126 (14.0%)	174 (14.4%)			
Other relative	3 (2.1%)	5 (3.0%)	20 (2.2%)	28 (2.3%)			
Total	142	168	900	1210	0.060	0.102	0.003

PTST- = Persistently negative tuberculin skin test; TSTC = Tuberculin skin test converter; TST + = tuberculin skin test positive at baseline.

*indicates *P* value that is significant after multiple testing correction.

**Table 3:** Clinical characteristics of index case by clinical group

	**PTST-**	**TSTC**	**TST+**	**All**	**χ **^**2 **^**across all 3 groups**	**χ **^**2 **^**for PTST- + TSTC vs. TST+**	**χ **^**2 **^**for PTST- vs. TSTC and TST+**
**Presence of Cavitary Disease**							
No	74 (53.2%)	75 (45.5%)	306 (34.7%)	455 (38.4%)			
Yes	65 (46.8%)	90 (54.5%)	576 (65.3%)	731 (61.6%)			
All	139	165	882	1186	<0.005*	<0.005*	<0.005*
**Baseline Extent of disease on chest x-ray**							
Normal	18 (12.9%)	9 (5.5%)	40 (4.5%)	67 (5.6%)			
Minimal	22 (15.8%)	22 (13.3%)	81 (9.2%)	125 (10.5%)			
Moderate	58 (41.7%)	76 (46.1%)	305 (34.6%)	439 (37.0%)			
Advanced	41 (29.5%)	58 (35.2%)	456 (51.7%)	555 (46.8%)			
All	139	165	882	1186	<0.005*	<0.005*	<0.005*
**High Index case Smear**							
None	4 (2.8%)	3 (1.8%)	14 (1.6%)	21 (1.7%)			
Scanty	5 (3.5%)	1 (0.6%)	11 (1.2%)	17 (1.4%)			
1+	13 (9.2%)	4 (2.4%)	21 (2.3%)	38 (3.1%)			
2+	31 (21.8%)	39 (23.2%)	89 (9.9%)	159 (13.1%)			
3+	89 (62.7%)	121 (72%)	765 (85%)	975 (80.6%)			
Total	142	168	900	1210	<0.005*	<0.005*	<0.005*
**Duration of Cough prior to treatment (days)**							
Mean (SD)	123.48 (155.876)	118.75 (124.032)	119.49 (119.597)		0.150	0.057	0.998
**Mycobacterial lineage**							
Lineage 4 Uganda	57 (46.3%)	71 (47.7%)	415 (54.0%)	543 (52.2%)			
Lineage 4 non_Uganda	49 (39.8%)	60 (40.3%)	280 (36.4%)	389 (37.4%)			
Lineage 3	17 (13.8%)	18 (12.1%)	74 (9.6%)	109 (10.5%)			
Total	123	149	769	1041	0.308	0.101	0.269

PTST- = Persistently negative tuberculin skin test; TSTC = Tuberculin skin test converter; TST + = tuberculin skin test positive at baseline; CI = confidence interval.

Lineage 4 is also known as Euro-American and Lineage 3 is also known as Central Asian [20].

*indicates *P* value that is significant after multiple testing correction.

### Ordinal regression analysis

Since we observed that the prevalence of individual risk factors was highest in TST+, followed by TSTC and then PTST-, we conducted an ordinal regression analysis (Table 4). Because of the parameterization of this model, an odds ratio > 1 is interpreted as increased “risk” of PTST- vs. TSTC, and an increased “risk” of TSTC vs. TST+. Thus, individuals age > 15 (OR = 0.43 *P* < 0.01) that slept in the same room as the index case (OR 0.66 *P* < 0.01) were less likely to be PTST-, and increasing number of windows in the home (beta = 1.10 *P* < 0.01) and greater number of people in the home (beta = 1.03 *P* < 0.01) were also associated with PTST-. The same interpretation can be made for TSTC versus baseline TST+. Thus, age, sleeping with the index case, ventilation, and crowding were independently associated with increased risk of TST conversion versus PTST-, and TST conversion versus pre-existing latent Mtb infection.

**Table 4:** Ordinal regression analysis of PTST- vs TSTC and TSTC vs TST+

	**Univariate model**			**Multivariable model**		
**Predictor**	**OR**	**95% CI**	***P***	**OR**	**95% CI**	***P***
Intercept 1	—	—		0.146	(0.090, 0.24)	
Intercept 2	—	—		0.400	(0.25, 0.65)	
Age 2–5 vs 0-2	1.43	(0.88, 2.32)	0.15	1.34	(0.82, 2.20)	0.24
Age 5–15 vs. 0-2	1.24	(0.82, 1.89)	0.31	1.04	(0.68, 1.60)	0.85
Age 15+ vs 0-2	0.553	(0.37, 0.84)	< 0.01	0.431	(0.28, 0.66)	< 0.01
No. of Windows	1.145	(1.09, 1.21)	< 0.01	1.097	(1.02, 1.18)	< 0.01
Sleep in Same Room	0.559	(0.43, 0.72)	< 0.01	0.66	(0.48, 0.89)	< 0.01
People in Home	1.070	(1.04, 1.10)	< 0.01	1.033	(1.00, 1.07)	< 0.01

PTST- = Persistently negative tuberculin skin test; TSTC = Tuberculin skin test converter; TST + = tuberculin skin test positive at baseline; CI = confidence interval. Univariate model results show results for each variable one at a time, while the multivariable model includes all variables. Model parameterized as logit(Pr(*y* ≤ *j*)) = α + **β***x* for *j* = 1 (PTST-), 2 (TSTC). Each OR shows the odds or PTST vs TSTC and also TSTC vs TST + .

### Risk score

**Figure 1:**
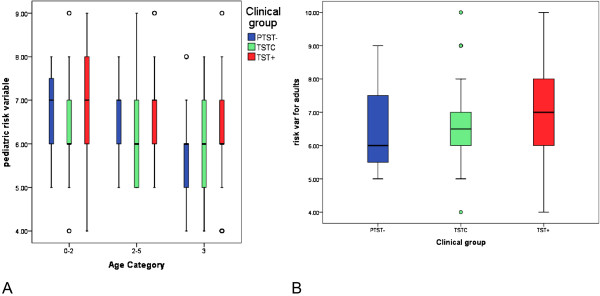
**Boxplot illustrating distribution of risk scores by clinical group in A) children < 15 years old (range 0 to 9) and B) adults (range 0 to 10).** PTST- (blue) = persistent TST negative, TSTC (green) = TST converter, and TST+ (red) = TST positive at baseline.

Next, we examined a risk score (Figure 1) [11], that was evaluated differently for children less than 15 years old (Additional file 1: Table S3) versus adults 15 years and older (Additional file 1: Table S4). Because our earlier analyses (Table 1) showed that age was a significant predictor of TST status and thus a confounder, we stratified by age group at the outset. This risk score demonstrated that all the children were highly exposed, with 95% having scores of 5 and above (Table 5). Furthermore, we observed no significant difference in the distribution of scores between the 3 clinical groups for the 0–2 and 2–5 age groups. In the 5–15 age group only, there were significant differences across the three groups (*P* = 0.009). In adults, there was no significant difference between the clinical groups in the distribution of the scores. Adults had a slightly lower degree of risk, with the percentage of individuals with scores of 6 and above ranging between 74.2% in PTST- to 90.8% in TSTC, though this difference was not statistically significant (Table 5 and Additional file 1: Table S4). When analyzing the risk score as a quantitative variable (Figure 1), the same conclusions can be drawn, with significant differences only occurring in the 5–15 age group (P = 0.006).

**Table 5:** Number of individuals by clinical group considered “high risk” according to Mandalakas et al. criteria

	**PTST-**	**TSTC**	**TST+**
2 years or less	19/19 (100%)	21/22 (95.5%)	101/103 (98.1%)
>2 and equal to 5	27/27 (100%)	24/24 (100%)	93/93 (100%)
>5 or less than 15	62/65 (95.4%)	45/47 (95.7%)	231/237 (97.5%)
>15	23/31 (74.2%)	68/75 (90.7%)	410/467 (87.8%)

“High risk” is defined as a pediatric risk score ≥5 out of 9, or an adult risk score ≥6 out of 10. Components of the risk score can be found in the Additional file 1, No statistically significant differences seen across clinical groups within age group (p > 0.05).

PTST- = Persistently negative tuberculin skin test; TSTC = Tuberculin skin test converter; TST + = tuberculin skin test positive at baseline.

Then, we stratified the adults into high and low risk groups according to this risk score, and re-analyzed the variables that were significant in our earlier analyses in Tables 2 and 3 but were not part of the risk score. Results are shown in Table 6. In the high-risk group, there were no longer differences between PTST- individuals and TSTC and TST+. Interestingly, this analysis demonstrated that the variables that were significant in the ordinal regression were no longer significant after accounting for an accumulation of other factors. Differences between individuals that were TST negative versus positive at baseline persisted (P < 0.0005, Table 6).

**Table 6:** Examination of “high risk” adults (age > 15) for key risk factors (N (%))

	**PTST-**	**TSTC**	**TST+**	**Comparison across all 3 groups**	**Comparison of PTST- vs TSTC + TST+**	**Comparison of PTST- and TSTC vs TST+**
***Characteristics of household***						
**Number of windows in house**						
Mean (SD)	3.48 (3.25)	3.10 (2.78)	1.93 (2.03)	< 0.0005	0.056	< 0.0005
**Number of rooms in house**						
Mean (SD)	4.26 (4.06)	3.87 (3.23)	2.77 (2.42)	0.002	0.096	< 0.0005
**# People in household/room**						
Mean (SD)	8.22 (7.30)	7.30 (5.88)	5.99 (3.93)	0.198	0.259	0.030
***Characteristics of index case***						
**Presence of cavitary disease**						
No	12 (54.5%)	28 (42.4%)	155 (38.4%)			
Yes	10 (45.5%)	38 (57.6%)	249 (61.6%)	0.290	0.181	0.231
**Baseline extent of disease on chest x-ray**						
Normal	3 (13.6%)	5 (7.6%)	13 (3.2%)			
Minimal	5 (22.7%)	7 (10.6%)	44 (10.9%)			
Moderate	7 (31.8%)	29 (43.9%)	145 (35.9%)			
Advanced	7 (31.8%)	25 (37.9%)	202 (50.0%)	0.069	0.085	0.033

“High risk” defined as a score ≥ 6 out of 10 on risk score (based on Mandalakas et al. criteria).

PTST- = Persistently negative tuberculin skin test; TSTC = Tuberculin skin test converter; TST + = tuberculin skin test positive at baseline.

## Discussion

Although many studies have evaluated risk factors for Mtb infection, few have looked at PTST- individuals. This large longitudinal study provides a unique opportunity to consider epidemiological factors associated with relative resistance to Mtb infection. Our study assessed household factors, individual factors, and index case characteristics that have been previously associated with presence of Mtb infection [2,4,5,11]. Several factors related to exposure intensity showed increasing prevalence from PTST- to TSTC to TST+. However, these risk factors do not exist in isolation, so the examination of a risk score such as that developed by Mandalakas et al. [11] may be more appropriate, as it reflects an accumulation of risk factors. We observed no significant differences in risk score distribution by TST status except in the 5–15 age group. Thus, when considering epidemiologic risk as an accumulation of risk factors, PTST- is not determined by decreased epidemiologic risk in young children and adults.

The 5–15 age group is the only group where differences were seen in the epidemiologic risk score between PTST-, TSTC and TST+. There are several possible explanations for this observation. First, children in this age group start attending school, and thus may start to have exposure to TB cases outside the home. Second, we have previously observed a significant difference in Mtb-specific interferon-γ responses in 5–15 year olds compared to younger children [22], reflecting the so-called “golden age of immunity”. The risk of progression from infection to disease is also lowest in the 5–10 year range [24], further reflecting a developing immune response.

One of the major characteristics of the PTST- group is a significantly younger age distribution that likely reflects less cumulative exposure to Mtb. Though sharing a bed was significantly different in the three clinical groups, this is likely because the youngest individuals are PTST-, and children are most likely to share a bed with their parent. When analyses were stratified by age group, factors such as sharing meals with the index case, severity of disease in the index case, crowding, and poor ventilation, that were previously statistically significant no longer were associated (data not shown). TST induration at baseline was also significantly different between PTST- and TSTC [15,25], suggesting that some individuals who were TST- at enrollment were progressing toward TST conversion, or perhaps that standard TST cutoff values resulted in misclassification.

A limitation of this analysis is that we did not conduct interferon-γ response assays (IGRA), on these study subjects to verify Mtb infection status. Many of these study participants were enrolled prior to the availability of the highly Mtb-specific IGRA. IGRAs were designed to avoid concerns of cross-reactivity with BCG and to allay concerns about boosting effects after repeated TSTs [19,26,27]. Indeed, our previous work shows that TSTC have a higher whole blood interferon-γ response at baseline than PTST- [15], so it is possible that an IGRA would have distinguished these two groups at baseline. However, the sensitivity [26,27] and cost-effectiveness [28] of IGRAs in TB-endemic settings is still a topic of debate. In addition, the risk score developed by Mandalakas et al. [11] can be used as a proxy for assessing infection, and the authors propose their risk score could be used in lieu of the TST or IGRA. However, this risk score has not been validated in independent studies, and in fact, our results suggest that the score does not associate with latent Mtb infection as was its developed purpose. In addition, the population studied by Mandalakas et al. was restricted to HIV negative individuals. Though our analyses included HIV positive individuals, their influence on the analysis was minimal, as only 1.7% of 0–2 year olds and 2.6% of 2–5 year olds were HIV positive. Furthermore, studies have shown that IGRA positivity is associated with indicators of recent and close Mtb exposure [26,27], which we have captured with the risk score. Another limitation is that there may be unmeasured exposure factors that could characterize PTST- individuals. We believe this is unlikely because our data collection was extensive.

## Conclusions

In summary, we examined a number of well-established variables associated with Mtb infection and disease, and found that a risk score consisting of factors associated with transmission of Mtb did not distinguish PTST- individuals from those who eventually convert their TST or were TST positive at baseline. There are likely host factors, such as host genetics [9] and immune response [15] that explain why these individuals remain relatively resistant to Mtb infection. Alternatively, unmeasured components of shared environment, such as nutrition [29,30] or quantity of Mtb bacilli in the air [31-33], may potentially explain why some individuals remain PTST-. Further study is needed to examine the combined influences of genetics, immunology, and nutrition on resistance to Mtb infection, as well as explore additional epidemiological factors.

## Abbreviations

BCG: Bacille Calmette-Guérin vaccine; HIV: Human immunodeficiency virus; Mtb: Mycobacterium tuberculosis; PPD: Purified protein derivative; PTST: Persistently tuberculin skin test negative; TB: Tuberculosis; TST: Tuberculin skin test; TSTC: Tuberculin skin test converter; TST+: Tuberculin skin test positive.

## Competing interests

There are no competing interests to report.

## Authors’ contributions

NM helped design the study, conducted the data analysis, conducted the literature of the review of the paper, and helped write all sections of the paper. SZ supervised the field activities and data quality assurance and control, and contributed to writing the paper. LLM supervised data quality and control of the study, created the analysis dataset, and edited all sections of the paper. MN helped supervise field activities of the study and helped write the paper. EMW conducted the lineage analysis. BAT contributed to the Results and Discussion sections of the paper. BO participated in field activities and helped write the paper. RPI conducted statistical analyses and helped write the paper. MLJ conducted the lineage analysis and supervised field activities. EM supervised the field activities and helped write the paper. HMK supervised the field activities and directed its implementation, and provided comments on the paper. WHB designed the study and directed its implementation, and helped write the paper. CMS designed the study and directed its implementation, designed the study’s analytic strategy, conducted data analyses, conducted the literature review, and wrote all sections of the paper. All authors read and approved the final manuscript.

## Pre-publication history

The pre-publication history for this paper can be accessed here:

http://www.biomedcentral.com/1471-2334/14/352/prepub

## Supplementary Material

Additional file 1 Supplemental material. Click here for file
