# Supplementary material for: Clinical and epidemiological characteristics of individuals resistant to M. tuberculosis infection in a longitudinal TB household contact study in Kampala, Uganda
Source: BMC Infect Dis. 2014 Jun 27;14:352. doi: 10.1186/1471-2334-14-352 (PMC4091673; doi:10.1186/1471-2334-14-352)
Supplement: Additional file 1 — Supplemental material. [file 1471-2334-14-352-S1.doc]

SUPPLEMENTAL MATERIAL

**Use of conservative tuberculin skin test cutoff**

Based on the observation that TSTC had a significantly different distribution of PPD induration compared to PTST- at baseline, we examined the use of a conservative 5 mm cutoff. Note that the 10 mm cutoff is more appropriate for TB-endemic settings and that it avoids potential misclassification due to reactivity to the BCG vaccine. Because 5 mm is the standard cutoff for children < 5 years old and for HIV infected individuals, we restricted this analysis to HIV sero-negative individuals 5 years old and older. In both the pediatric and adult groups, we observed a statistically significantly higher proportion of eventual TSTC with a PPD > 5 mm induration compared to PTST- (Supplemental Table 1). Our previous work (Whalen et al. 2006, Mahan et al. 2012) showed this same trend and we hypothesize that individuals who eventually convert their TST are in the process of that conversion at the time that we enroll the household. Because the 10 mm cutoff is more appropriate in our research setting, we used it in further analyses.

**Supplemental Table 1. Baseline tuberculin skin test positivity in PTST- and TSTC using 5 mm cutoff (HIV uninfected individuals only)^1^**

| **Age group** | **Clinical group** | **PPD < 5 mm**  **N (% within clinical group)** | **PPD ≥ 5 mm**  **N (% within clinical group)** |
| --- | --- | --- | --- |
| 5-15^2^ | PTST- | 58 (95.1%) | 3 (4.9%) |
|  | TSTC | 36 (76.6%) | 11 (23.4%) |
| 15+^3^ | PTST- | 25 (100%) | 0 (0%) |
|  | TSTC | 46 (64.8%) | 25 (35.2%) |

PTST- = Persistently negative tuberculin skin test; TSTC= Tuberculin skin test converter

^1^ All Individuals that were HIV negative and age 5 years old and above that had a PPD > 10 mm at baseline were classified as TST positive and thus not relevant to this analysis

^2^Fisher’s exact p=0.008

^3^ Fisher’s exact p=0.007

**Supplemental Table 2. Comparison of persistent TST negative individuals observed after 12 versus 24 months of follow-up**

|  | **Ptst-12 months** | | **Ptst- 24 months** | **All** | **Comparison of two groups** |
| --- | --- | --- | --- | --- | --- |
| **Age** |  | |  |  |  |
| 2 years or less | 8 (25.8%) | | 11 (9.9%) | 19 (13.4%) |  |
| >2 and equal to 5 | 8 (25.8%) | | 19 (17.1%) | 27 (19%) |  |
| >5 or less than 15 | 9 (29.0%) | | 56 (50.5%) | 65 (45.8%) |  |
| >15 | 6 (19.4%) | | 25 (22.5%) | 31 (21.8%) |  |
| All | 31 | | 111 | 142 | 0.044 |
| **Sex** |  | |  |  |  |
| Female | 18 (58.1%) | | 55 (49.5%) | 73 (51.4%) |  |
| Male | 13 (41.9%) | | 56 (50.5%) | 69 (48.6%) |  |
| All | 31 | | 111 | 142 | 0.402 |
| **HIV status** | | | | | |
| Negative | 27 (90.0%) | | 100 (91.7%) | 127 (91.4%) |  |
| Positive | 3 (10.0%) | | 9 (8.6%) | 12 (8.6%) |  |
| All | 30 | | 109 | 139 | 0.763 |
| **Presence of BCG scar** | | | | | |
| No | 4 (12.9%) | | 23 (20.7%) | 27 (19%) |  |
| Yes | 22 (23.7%) | | 71 (64.0%) | 93 (65.5%) |  |
| Uncertain | 5 (16.1%) | | 17 (15.3%) | 22 (15.5%) |  |
| All | 31 | | 111 | 142 | 0.615 |
| **Share bed with index case** | | | | | |
| No | 28 (90.3%) | | 94 (84.7%) | 122 (85.9%) |  |
| Yes | 3 (9.7%) | | 17 (15.3%) | 20 (14.1%) |  |
| All | 31 | | 111 | 142 | 0.425 |
| **Share room with index case** | | | | | |
| No | 15 (48.4%) | | 50 (45.0%) | 65 (45.8%) |  |
| Yes | 16 (51.6%) | | 61 (55.0%) | 77 (54.2%) |  |
| All | 31 | | 111 | 142 | 0.741 |
| **Share meals with index case** | | | | | |
| No | 5 (16.1%) | | 9 (8.1%) | 14 (9.9%) |  |
| Yes | 26 (83.9%) | | 102 (91.9%) | 128 (90.1%) |  |
| All | 31 | | 111 | 142 | 0.185 |
| **Type of House** | | | | | |
| Not muzigo | 20 (64.5%) | | 55 (49.5%) | 75 (52.8%) |  |
| Muzigo | 11 (35.5%) | | 56 (50.5%) | 67 (47.2%) |  |
| All | 31 | | 111 | 142 | 0.14 |
| **Presence of Cavitary Disease** | | | | | |
| No | 15 (51.7%) | | 59 (53.6%) | 74 (53.2%) |  |
| Yes | 14 (48.3%) | | 51 (46.4%) | 65 (46.8%) |  |
| All | 29 | | 110 | 139 | 0.854 |
| **Baseline Extent of disease on chest x-ray** | | | | | |
| Normal | 5 (17.2%) | | 13 (11.8%) | 18 (12.9%) |  |
| Minimal | 3 (10.3%) | | 19 (17.3%) | 22 (15.8%) |  |
| Moderate | 12 (41.4%) | | 46 (41.8%) | 58 (41.7%) |  |
| Advanced | 9 (31.0%) | | 32 (29.1%) | 41 (29.5%) |  |
| All | 29 | | 110 | 139 | 0.742 |
| **High Index case Smear** | | | | | |
| None | 1 (3.2%) | | 3 (2.7%) | 4 (2.8%) |  |
| Scanty | 1 (3.2%) | | 4 (3.6%) | 5 (3.5%) |  |
| 1+ | 2 (6.5%) | | 11 (9.9%) | 13 (9.2%) |  |
| 2+ | 3 (9.7%) | | 28 (25.2%) | 31 (21.8%) |  |
| 3+ | 24 (77.4%) | | 65 (58.6%) | 89 (62.7%) |  |
| Total | 31 | | 111 | 142 | 0.354 |
| **Frequency of contact with index case (day/week)** | | | | | |
| <1 days | | 0 (0.0%) | 1 (0.9%) | 1 |  |
| 1-3 days | | 2 (6.5%) | 4 (3.6%) | 6 |  |
| 4-6 days | | 2 (6.5%) | 5 (4.5%) | 7 |  |
| daily | | 27 (87.1%) | 101 (91.0%) | 128 |  |
| Total | | 31 | 111 | 142 | 0.808 |
| **Time spent with index case (hours)** | | | | | |
| <1 hr | | 0 (0.00%) | 3 (2.7%) | 3 |  |
| 2-6 hr | | 2 (6.5%) | 15 (13.6%) | 17 |  |
| 7-12 hr | | 17(54.8%) | 53 (48.2%) | 70 |  |
| 13-18 hr | | 6 (19.4%) | 23 (20.9%) | 29 |  |
| >18 hr | | 6 (19.4%) | 16 (14.5%) | 22 |  |
| Total | | 31 | 110 | 141 | 0.648 |
| **Relationship to index case** | | | | | |
| Spouse | | 0 (0.0%) | 6 (5.4%) | 6 (4.2%) |  |
| Parent | | 0 (0.0%) | 3 (2.7%) | 3 (2.1%) |  |
| Sibling | | 7 (22.6%) | 16 (14.4%) | 23 (16.2%) |  |
| Avuncular | | 3 (9.7%) | 21 (18.9%) | 24 (16.9%) |  |
| Child | | 17 (54.8%) | 42 (37.8%) | 59 (41.5%) |  |
| Grandparent/Child | | 2 (6.5%) | 1 (0.9%) | 3 (2.1%) |  |
| Unrelated | | 2 (6.5%) | 19 (17.1%) | 21 (14.8%) |  |
| Other relative | | 0 (0.0%) | 3 (2.7%) | 3 (2.1%) |  |
| Total | | 31 | 111 | 142 | 0.081 |
| **PPD at baseline (mm)** | | | | | |
| Mean (SD) | | 0.49 (1.676) | 0.25 (0.961) |  | 0.732 |
| **Number of windows in house** | | | | | |
| Mean (SD) | | 2.55 (2.631) | 2.61 (2.823) |  | 0.986 |
| **Number of rooms in house** | | | | | |
| Mean (SD) | | 3.26 (2.435) | 3.49 (3.371) |  | 0.739 |
| **# People in household / room** | | | | | |
| Mean (SD) | | 6.55 (2.541) | 8.15 (5.727) |  | 0.477 |
| **Number of TB cases in home** | | | | | |
| Mean (SD) | | 1.74 (1.154) | 1.65 (0.931) |  | 0.92 |

PTST- = Persistently negative tuberculin skin test; TSTC= Tuberculin skin test converter; TST+ = tuberculin skin test positive at baseline; CI = confidence interval

Our objective was to examine PTST- individuals with only 12 months versus 24 months of follow-up (all age groups). We examined the same variables as in Tables 2 and 3, and found that with the exception of age group (*p*=0.049), none of the comparisons were nominally significant. There were fewer young children (0-2 and 2-5) (9.9% and 17.1%, respectively) with 24 months of follow-compared to those with 12 months of follow-up (25.8% and 25.8%, respectively). Young children were more likely to be lost to follow-up since mothers voiced concerns about longitudinal study visits and/or blood draws. These results suggest that epidemiologically, with less follow-up do not differ, and perhaps one year of follow-up is sufficient to identify this clinical phenotype.

**Components of risk score**

*Pediatric risk score (as in Mandalakas et al. 2012)*

Is the index case the child’s mother?

Does the index case sleep in the same bed as the child?

Does the index case sleep in the same room as the child?

Is the index case coughing?

Does the index case have reported pulmonary TB?

Does the index case have smear-positive sputum?

Does the index case live in the same household as the child?

Does the index case see the child every day?

Is there more than one adult TB case in the child’s household?

*Item included in Mandalakas’ score that we did not collect:*

Is the index case the child’s caregiver?

*Adult risk score*

Is the index case the adult’s spouse?

Is the adult the index case’s primary caregiver?

Does the index case sleep in the same bed as the adult?

Does the index case sleep in the same room as the adult?

Is the index case coughing?

Does the index case have reported pulmonary TB?

Does the index case have smear-positive sputum?

Does the index case live in the same household as the adult?

Does the index case see the adult every day?

Is there more than one adult TB case in the adult’s household?

Supplemental Table 3. Risk score in pediatric age groups

|  | **0-2 years old** | | | **2-5 years old** | | | **5-15^a^ years old** | | |
| --- | --- | --- | --- | --- | --- | --- | --- | --- | --- |
| **Score^b^** | **PTST-**  **N=19** | **TSTC**  **N=22** | **TST+**  **N=103** | **PTST-**  **N=27** | **TSTC**  **N=24** | **TST+**  **N=93** | **PTST-**  **N=65** | **TSTC**  **N=47** | **TST+**  **N=237** |
| 4 | 0(0.0) | 1(4.5) | 2(1.9) |  |  |  | 3(4.6) | 2(4.3) | 6(2.5) |
| 5 | 3(15.8) | 4(18.2) | 8(7.8) | 6(22.2) | 10(41.7) | 12(12.9) | 24(36.9) | 11(23.4) | 36(15.2) |
| 6 | 6(31.6) | 9(40.9) | 32(31.1) | 6(22.2) | 7(29.2) | 31(33.3) | 27(41.5) | 16(34.0) | 119(50.2) |
| 7 | 5(26.3) | 4(18.2) | 26(25.2) | 10(37.0) | 3(12.5) | 32(34.4) | 10(15.4) | 12(25.5) | 81(24.9) |
| 8 | 5(26.3) | 3(13.6) | 29(28.2) | 5(18.5) | 3(12.5) | 17(18.13 | 1(1.5) | 6(12.8) | 16(6.8) |
| 9 | 0(0.0) | 1(4.5) | 6(5.8) | 0(0.0) | 1(4.2) | 1(1.1) | 0(0.0) | 0(0.0) | 1(0.4) |
| Mean (SD) | 6.63 (1.07) | 6.31 (1.21) | 6.87 (1.14) | 6.51 (1.05) | 6.08 (1.21) | 6.61 (0.97) | 5.72 (0.83) | 6.19 (1.08) | 6.19 (0.88) |

PTST- = Persistently negative tuberculin skin test; TSTC= Tuberculin skin test converter; TST+ = tuberculin skin test positive at baseline, SD = standard deviation

^a^ Categorical data analysis comparison across all 3 groups *p*=0.009, comparison by TST status at baseline (PTST- + TSTC vs TST+) *p*=0.014, comparison of PTST- vs others *p*=0.004. Analysis as a quantitative variable comparison across all 3 groups P=0.001, comparison by TST status at baseline (PTST- + TSTC vs TST+) *p*=0.006, comparison of PTST- vs others *p*< 0.0005 (age 5-15 only).

^b^ Pediatric risk score only goes up to 9, but adult score goes up to 10

Supplemental Table 4. Risk score in adults age 15 and above

| **Score** | **PTST-**  **N=31** | **TSTC**  **N=75** | **TST+**  **N=467** |
| --- | --- | --- | --- |
| 4 | 0(0.0) | 1(1.3) | 8(1.7) |
| 5 | 8(25.8) | 6(8.0) | 49(10.5) |
| 6 | 11(35.5) | 31(41.3) | 139(29.8) |
| 7 | 4(12.9) | 22(29.3) | 120(25.7) |
| 8 | 4(12.9) | 6(8.0) | 65(13.9) |
| 9 | 4(12.9) | 7(9.3) | 70(15.0) |
| 10 | 0(0.0) | 2(2.7) | 16(3.4) |
| Mean (SD) | 6.51 (1.36) | 6.73 (1.22) | 6.98 (1.39) |

PTST- = Persistently negative tuberculin skin test; TSTC= Tuberculin skin test converter; TST+ = tuberculin skin test positive at baseline, SD = standard deviation
